# Reentry and Ectopic Pacemakers Emerge in a Three-Dimensional Model for a Slab of Cardiac Tissue with Diffuse Microfibrosis near the Percolation Threshold

**DOI:** 10.1371/journal.pone.0166972

**Published:** 2016-11-22

**Authors:** Sergio Alonso, Rodrigo Weber dos Santos, Markus Bär

**Affiliations:** 1 Department of Physics, Universitat Politècnica de Catalunya, Barcelona, Spain; 2 Physikalisch-Technische Bundesanstalt, Berlin and Braunschweig, Germany; 3 Graduate Program in Computational Modeling, Federal University of Juiz de Fora, Juiz de Fora, Brazil; Universiteit Gent, BELGIUM

## Abstract

Arrhythmias in cardiac tissue are generally associated with irregular electrical wave propagation in the heart. Cardiac tissue is formed by a discrete cell network, which is often heterogeneous. Recently, it was shown in simulations of two-dimensional (2D) discrete models of cardiac tissue that a wave crossing a fibrotic, heterogeneous region may produce reentry and transient or persistent ectopic activity provided the fraction of conducting connections is just above the percolation threshold. Here, we investigate the occurrence of these phenomena in three-dimensions by simulations of a discrete model representing a thin slab of cardiac tissue. This is motivated (i) by the necessity to study the relevance and properties of the percolation-related mechanism for the emergence of microreentries in three dimensions and (ii) by the fact that atrial tissue is quite thin in comparison with ventricular tissue. Here, we simplify the model by neglecting details of tissue anatomy, e. g. geometries of atria or ventricles and the anisotropy in the conductivity. Hence, our modeling study is confined to the investigation of the effect of the tissue thickness as well as to the comparison of the dynamics of electrical excitation in a 2D layer with the one in a 3D slab. Our results indicate a strong and non-trivial effect of the thickness even for thin tissue slabs on the probability of microreentries and ectopic beat generation. The strong correlation of the occurrence of microreentry with the percolation threshold reported earlier in 2D layers persists in 3D slabs. Finally, a qualitative agreement of 3D simulated electrograms in the fibrotic region with the experimentally observed complex fractional atrial electrograms (CFAE) as well as strong difference between simulated electrograms in 2D and 3D were found for the cases where reentry and ectopic activity were triggered by the micro-fibrotic region.

## Introduction

The synchronous contraction of the heart is a consequence of the highly organized propagation of the membrane action potential through the whole cardiac tissue. Several cardiac arrhythmias correspond to conditions where this synchronization fails and cardiac contraction is compromised. One of the most relevant arrhythmias is fibrillation which corresponds to a state where the contraction of many cardiac myocytes are strongly desynchronized in the atria, i.e. atria fibrillation, or, with more dangerous consequences, in the ventricles, i.e. ventricular fibrillation. Reentry is a circulating action potential wave, and believed to be a necessary but not a sufficient condition for fibrillation. Fibrillation is often attributed to a succession of multiple breakups and pairwise annihilations of spiral waves.

Spiral generation and spiral breakup [1–4] have been extensively studied by computational modeling, for reviews see [5, 6]. The generation of a spiral wave may result from the impact of an ectopic beat: an additional, premature stimulus (S2) which generates a second wave that interacts with the sinus wave (S1) giving rise to a spiral wave [5]. A second common mechanism for spiral wave generation is cardiac alternans, the alternation of short and large pulses in response to a high frequency pacing [6, 7].

Even though atrial fibrillation (AF) is the most common type of sustained cardiac arrhythmia [8], the mechanisms that trigger it are still poorly understood. One hypothesis supported by clinical studies [9] and animal experiments [10] is that AF can be initiated by one or more regions that generate ectopic beats. For example, six consecutive ectopic beats separated by 130 ms may induce atrial fibrillation [11]. Structural heterogeneities may also produce persistent atrial fibrillation [12].

In this work, we study how an heterogeneous region with the presence of both full-connected and non-conducting (fibrosis) areas can generate microreentry in 3D computational models of cardiac tissue. This type of fibrosis is known as microfibrosis due to the microscopic scale of the heterogeneities [13–15].

In 2007, Spach et al. [16] could reproduce their original experiments [17] using a 2D *in-silico* model that also accounted for fibrosis. A single cycle of microreentry was generated after using a standard S1–S2 protocol. They concluded that fibrosis increases the vulnerability to fast pacing. Similar results using multiple stimulus protocols were obtained by different groups [16, 18–22] and confirmed that fibrosis or other micro-structural heterogeneities may facilitate the initiation of reentries and help to sustain reentry or fibrillation. The use of the words facilitate and help appears naturally, since it is well known that S1–S2 or fast-pacing protocols can *per se* generate reentries. Therefore, there exists a complicated interaction among the different waves (S1–S2) and the heterogeneity of the tissue (fibrosis).

However, it was only recently shown [23, 24] that we can generate microreentries and ectopic pacemakers in 2D computer simulations of cardiac electrophysiology without the use of a second premature stimulus (S2) [16] or fast pacing [18]. If only a single stimulus is used, the usual interactions between subsequent waves are not present; and therefore, one can relate the genesis of the microrentries inside the fibrosis to the specific heterogeneity of the tissue, i.e. to its micro-structure rather than to functional features (like wave interactions).

The fibrosis forms a maze where waves fractionate and follow zig-zag pathways. Therefore, the topology of the maze is crucial for the emergence of a reentry inside the fibrotic region [23, 24]. This wave activity may continuously or transiently generate ectopic beats when it crosses the border of the microfibrosis region [18] into the healthy tissue areas. Such zig-zag dynamics was previously observed in experimental studies [25].

A straightforward approach to the modeling of microfibrosis is the use of simple 2D discrete and isotropic models of cardiac tissue, wherein a prescribed percentage of connections between neighboring myocytes is randomly removed [23, 26]. The probability of microreentry depends on the degree of fibrosis specified by the fraction *ϕ* of non-conducting links between neighboring cells in the tissue. By analyzing the probability distribution of tissues with reentry as a function of *ϕ*, it was found [23] that the maximum of this distribution was close to the percolation threshold *ϕ*_*c*_ [27, 28] of the conducting links in the associated discrete network. The value of *ϕ*_*c*_ provides a reference parameter that depends only on the topology, and, therefore, on the spatial dimension of the lattice used in the simulation. A similar relation was verified later using two-dimensional microscopic anisotropic models of the tissue together with more detailed physiological descriptions [24]. These results indicate that the degree of fibrosis as well as the topology of connections between myocytes are important factors when evaluating the pro-arrhythmic behavior of a particular region of the heart.

In recent years, related studies were carried out by Christensen et al. [29] for an anisotropic tissue model, by Kazbanov et al. [30] for tissues with spatially varying degrees of microfibrosis and by Vigmond et al. [19] for the modeling of complex fractionated atrial electrograms (CFAE) and reentry in a fibrosis model based on imaging data.

Propagation near fibrotic area has been associated with CFAE, in both animal [31] and clinical studies [32]. These findings have remarkably changed the management and prognostics of patients with atrial fibrillation via a new procedure of catheter ablation of atrial fibrillation that is guided by CFAE mapping [33]. Recent clinical studies of guided CFAE ablation revisited the benefits and risks of this procedure and clearly suggest that further investigation on this topic is required [34–36]. Different computer simulations have also already given valuable insights into how the microstructure of cardiac tissue, specifically microfibrosis, and CFAE are related [13, 15].

Here, we extend our studies with 2D simulations presented before in [23, 24] to 3D simulations of thin cardiac tissue represented by a discrete model composed of up to twenty, two-dimensional layers arranged in a cubic lattice with an embedded region of microfibrosis that will be shown: i) to reproduce microreentry inside the microfibrosis tissue, ii) to form an ectopic pacemaker and iii) to reproduce qualitatively the signature of CFAE.

In addition, the simulations permit to quantify the influence in the activity of the ectopic pacemaker of important physiological parameters such as electrical remodeling, the fraction of fibrosis (i. e. the fraction of broken links), the size of the micro-fibrotic region and the tissue thickness.

Many simulations of the atria are based on the monolayer hypothesis [11, 37], however, tissue thickness of the atria is known to vary between 0.5 and 3 *mm* [38]. In addition, some recent studies have suggested the possibility of transmural reentries in the atria [39], a phenomenon that could only be captured with a 3D model. Here, we investigate how the number of layers in the third dimension influences the occurrence of microreentries in a fibrotic region of the atria.

Electrophysiological properties such as APD and conduction velocity (CV) vary substantially within the heart. In addition, different pathologies, such as atrial fibrillation, ischemia or a substantial increase of myocyte to fibroblast coupling, may significantly decrease the values of both APD and CV [11, 40–43]. To assess how different electrophysiological features influences the probability to generate microreentries in a fibrotic region we test three levels of electrophysiological remodeling: normal remodeling (APD = 135ms, CV = 44 cm/s); intermediate remodeling (APD = 120ms, CV = 39 cm/s), and strong remodeling (APD = 76ms, CV = 34 cm/s).

Finally, the degree, type and pattern of fibrosis varies considerably for different kinds of diseases or during different stages of one particular pathology [44]. Therefore, we have investigated how the percentage of fibrosis, *ϕ*, and the size of a fibrotic region influence the probability to generate microreentries and create ectopic pacemakers in cardiac tissue.

## Materials and Methods

### Continuous model of cardiac tissue

The propagation of the action potential is described by the cable equation [45]. The equation relates the variation of the membrane potential (*V*) with the total ion current through the ion channels across the membrane (*I*), and the conduction of potential along the cell membrane. The cable equation reads:

$$\begin{array}{ccc} \frac{\partial V}{\partial t} & = & -I+\vec{\nabla }\cdot \left(D\vec{\nabla }V\right), \end{array}$$ (1)

where *D* = 1 *cm*^2^/*s* is the effective diffusion coefficient of the action potential [6]. The above equation is solved in its discrete form, as will be explained in the next sections. Moreover, homogeneous Neumann boundary conditions are used. We do not consider anisotropy or any detailed geometry and anatomy of the tissue to avoid extensive calculations. This amounts to a qualitative description of the tissue which nevertheless captures the main properties of the dynamics as will be demonstrated below.

The total current *I* is the sum of different types of ion currents *I* = ∑_*i*_ *I*_*i*_. The number and form of these currents is determined by the specific ionic model employed [46]. Here, we use a simplified and generic cardiac model, which consists of only three ion currents [4, 47]:

$$\begin{array}{ccc} \frac{\partial V}{\partial t} & = & -\left(I_{fi}+I_{so}+I_{si}\right)+\vec{\nabla }\cdot \left(D\vec{\nabla }V\right), \end{array}$$ (2)

where the currents do not exactly correspond to an explicit ion channel, but are controlled by parameters which can be fitted to particular experimental measurements or to more detailed models. The ionic currents read:

$$\begin{array}{ccc} I_{fi} & = & \frac{-vp\left(V-V_{c}\right)\left(1-V\right)}{\tau_{d}},\\ I_{so} & = & \frac{V(1-p)}{\tau_{o}}+\frac{p}{\tau_{r}},\\ I_{si} & = & \frac{-w(1+tanh\left(k\left(V-V_{c}^{si}\right)\right))}{2\tau_{si}}, \end{array}$$ (3)

corresponding, to the fast inward (*I*_*fi*_), slow outward (*I*_*so*_) and slow inward (*I*_*si*_) currents. These currents are controlled by the gating variables *v* and *w*. The evolution of such gate variables depends on the membrane potential:

$$\begin{array}{ccc} \frac{\partial v}{\partial t} & = & \frac{\left(1-p)(1-v\right)}{\tau_{v}^{-}\left(V\right)}-\frac{pv}{\tau_{v}^{+}},\\ \frac{\partial w}{\partial t} & = & \frac{\left(1-p)(1-w\right)}{\tau_{w}^{-}}-\frac{pw}{\tau_{w}^{+}}; \end{array}$$ (4)

where $\tau_{v}^{-}(V)=(1-q)\tau_{v1}^{-}+q\tau_{v2}^{-}$ with *p* = *θ*(*V* − *V*_*c*_) and *q* = *θ*(*V* − *V*_*v*_), where *θ*(*x*) is the Heaviside step function. We have used the same parameter values as described before in [23]. One of the main advantages of using this simplified and generic cardiac cell model is the ability to test different scenarios in terms of electrophysiological remodeling via the modification of a single parameter of the model, *τ*_*d*_.

We employ finite differences for the spatial discretization (Δ*x* = 100 *μm*) and a second order Runge-Kutta method for the temporal integration of the equations (Δ*t* = 0.0165 *ms*) for the numerical integration of Eqs (2–4).

### Excitability and level of remodeling

Electrophysiological properties such as APD and CV vary substantially within the heart. In addition, under different pathologies, such as atria fibrillation, ischemia or a substantial increase of myocyte to fibroblast coupling, both APD and CV values can be significantly decreased. For instance, whereas APD values can be as short as 140 ms in healthy atria, it is reduced to 56 ms under chronic atrial fibrillation [11, 40, 41]. Likewise, CV values may be as slow as 44 cm/s within normal atrial, whereas under chronic atrial fibrillation CV can be even slower, with reported values at 37 cm/s [11, 40–43].

Numerical simulation of electrical remodeling has been used in many studies of atria fibrillation. Action potential duration and conduction velocities decreased under remodeling to: *APD* = 100 *ms* and *CV* = 66 *cm*/*s* in [48], *APD* = 144 *ms* and *CV* = 54 *cm*/*s* [38], and *APD* = 75 *ms* and *CV* = 61 *cm*/*s* [11]. AP remodeling can be due to completely different reasons, and the level of remodeling depends not only on the type of pathology but also on its stage.

In this work, we use a simplified approach to assess how different electrophysiological features influence the probability to generate microreentries in a fibrotic region. Despite the multitude of possibilities in terms of APD and CV values reported, we simulate only three levels of electrophysiological remodeling: normal remodeling (APD = 135ms, CV = 44 cm/s); intermediate remodeling (APD = 120ms, CV = 39 cm/s), and strong remodeling (APD = 76ms, CV = 34 cm/s).

**Fig 1:**
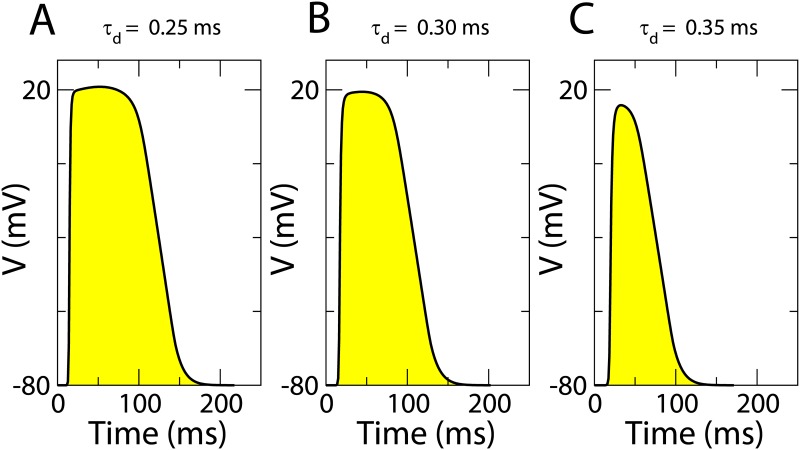
Transmembrane voltage evolution for different remodeling levels. Shape of the action potential duration for different values of *τ*_*d*_ employed in the three-dimensional simulations: *τ*_*d*_ = 0.25 *ms* (A), *τ*_*d*_ = 0.30 *ms* (B), and *τ*_*d*_ = 0.35 *ms* (C). Parameters used are: $\tau_{v}^{+}=$ 3.33*ms*, $\tau_{v1}^{-}$ = 19.6 *ms*, $\tau_{v2}^{-}$ = 1000 *ms*, $\tau_{w}^{+}$ = 667 *ms*, $\tau_{w}^{-}$ = 11 *ms*, *τ*_*o*_ = 8.3 *ms*, *τ*_*r*_ = 50 *ms*, *τ*_*si*_ = 45 *ms*, *k* = 10, $V_{c}^{si}$ = 0.85, *V*_*c*_ = 0.13 and *V*_*v*_ = 0.055, for more details see [4]. These parameter values are kept constant for the rest of simulations.

These three cases of remodeling were simulated by using different values of the parameter *τ*_*d*_ of the Fenton-Karma model [47]: 0.25 ms, 0.30 ms and 0.35 ms. The action potential duration (APD) and the conduction velocity (CV) of our simplified cell model decrease with the parameter *τ*_*d*_, and consequently, the wave length (*WL*) given by the product of these two quantities (*WL* = *CV* ⋅ *APD*) also decreases. Thus, waves are smaller and slower for larger values of *τ*_*d*_, implying that the excitability decreases with this parameter. Therefore we make the qualitative relation between decreased excitability and electrical remodeling, i.e. higher values of *τ*_*d*_ corresponds to higher level of remodeling, as presented in Fig 1.

### Discrete heterogeneous cardiac model

Randomly distributed inactive gap junctions, inert tissue cells among the cardiac muscle cells or the presence of unexcitable cells, e.g. fibroblast, in the tissue break the homogeneous propagation of action potential through the cardiac tissue. The resulting waves are expected to be corrugated and noisy. We model this disruption in the propagation by the introduction of random heterogeneities in the network of cells using the connectivity parameter *η*_*ij*_:

$$\begin{array}{ccc} \frac{\partial V_{i}}{\partial t} & = & -\left(I_{fi}+I_{so}+I_{si}\right)+\xi \sum_{j}^{N}\eta_{ij}\left(V_{j}-V_{i}\right), \end{array}$$ (5)

where *ξ* = *D*/*ℓ*^2^ with *ℓ* = 100 *μm*. If the connectivity parameter *η*_*ij*_ = 1 for all *i* and *j* we recover the homogeneous discrete network limit of Eq (2). The connection between two cells (*i* and *j*) is removed (*η*_*ij*_ = 0) with a certain probability *ϕ*. These abnormal connections are randomly scattered in the tissue. Our approach is a simple and qualitative model of reactive interstitial fibrosis [49].

### Definition and calculation of the percolation threshold

The increase of the fraction of non-conducting links leads to isolated regions confined by removed links. Below a particular fraction of *ϕ*_*c*_ the network exhibits a percolation of conducting links [50]. Wave propagation is not possible for fractions above this percolation threshold. For *ϕ* just below the value of *ϕ*_*c*_, the effective medium approach [51] that enables modelers to replace heterogeneous discrete models by homogeneous continuum models breaks down and a single excitation wave may break into pieces and produce reentries and persistent irregular activity [23].

The percolation threshold depends on several factors including the spatial dimension of the system. The value of the fraction *ϕ* that produces conduction block in a particular two-dimensional lattice may permit propagation in a different lattice with higher connectivity or in the corresponding 3D medium. Therefore, it is not clear that previous results in 2D can be extrapolated to 3D. Here, we want to evaluate this transition from 2D to 3D systems by using a discrete model of up to 20 layer thickness defined on a cubic lattice. The layers of this model correspond to the square lattices employed in early studies in 2D [23].

**Fig 2:**
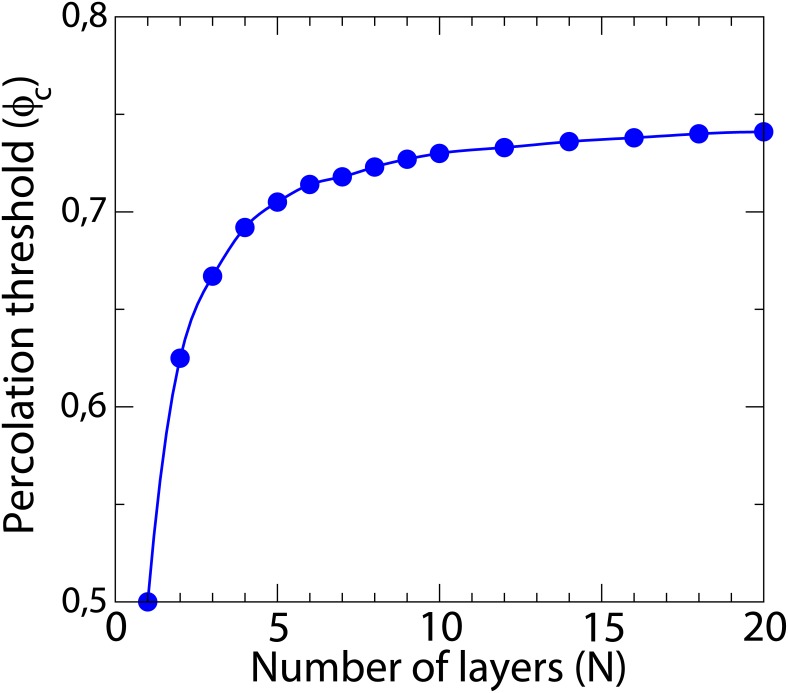
Percolation threshold for different slab thickness. Dependence of the percolation threshold in a slab of tissue for different thickness. For each thickness *N* we generate 100 realizations for each value of *ϕ* and check the probability of finding a pathway from one side of the system to the other. After a fitting process, we evaluate the fraction *ϕ*_*c*_ which correspond to a probability of 50%. The size of the system is 4 *cm* × 4 *cm* × *N* *layers*, where each layer corresponds to 0.01 *cm*.

We systematically increase the number of layers (N) in a three-dimensional model starting from a monolayer, i.e. the two-dimensional system, assuming a layer thickness of 100 *μ*m. Altogether, we vary from *N* = 1 (monolayer, 2D) to *N* = 20 layers (3D tissue with a thickness of 2 mm). For these parameters the percolation threshold can be easily calculated from one hundred randomly generated networks for different values of *ϕ* [23]. The change of the percolation threshold is plotted in Fig 2.

### Simulation protocol

We consider that a small cylindrical region in the center of our simulation domain represents the fibrotic region and it is strongly heterogeneous. Inside this fibrotic region, a fraction *ϕ* of connections are non-conducting (*η*_*ij*_ = 0). Outside this fibrotic region all the connections between neighboring myocytes are normal and homogeneous (*η*_*ij*_ = 1). In addition, we model electrical remodeling varying the excitability of the fibrotic region, we set *τ*_*d*_ to $\tau_{d}^{o}$, where $\tau_{d}^{o}$ can be 0.25 *ms*, 0.30 *ms* or 0.35 *ms*.

We evaluate the interaction between the wave propagating in an homogeneous region of the tissue with the fibrotic region with a fraction *ϕ* of non-conducting links. A single excitation wave of action potential is initially induced in a corner of the three-dimensional slab. The action potential propagates through the medium till it interacts with the fibrotic region. Depending on the values of *ϕ* and *ϕ*_*c*_ (percolation threshold for the slab used in simulation, see Fig 2) three behaviors are observed: For low fraction (*ϕ* ≪ *ϕ*_*c*_) the wave propagates inside the fibrotic region and deforms, and although propagation inside is slower than outside, wave propagation is stable. For large fraction (*ϕ* ≫ *ϕ*_*c*_) the wave does not enter into the fibrotic region and propagates only around this region. For intermediate fraction (*ϕ* ∼ *ϕ*_*c*_) the wave propagates in the fibrotic region, however the propagation inside is highly irregular. We evaluate the different dynamics by simulating the activity during 1500 *ms*. The resulting numerical simulations are discussed in the next section.

### Fibrotic border effects

We incorporate a fibrotic border (FB) that surrounds the region with microfibrosis. This border region models a smooth transition between non-fibrotic and fibrotic tissue in comparison with a stiff transition employed in our previous models [23, 24].

**Fig 3:**
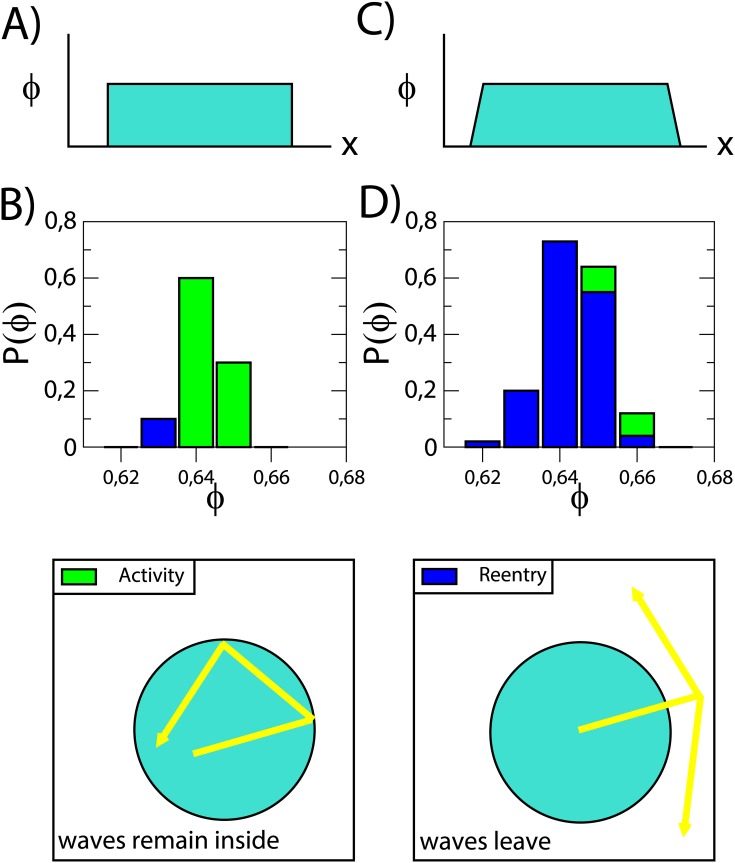
Effects of the fibrotic border in the fibrotic area. Sketch of the fibrotic region without (a) and with (c) fibrotic border and probability of reentry without (b) and with (d) fibrotic border for *N* = 3 in a system 4 × 4 × 0.03 *cm*^3^ and *R*_0_ = 1.40 *cm* for a total time of simulation of 1500 *ms*. Two types of dynamics are observed: reentry outside of the fibrotic region, *Reentry* (blue) or activity restricted to the interior of the fibrotic region, *Activity* (green).

First, we compare the two different borders: 1) a localized fibrosis without FB, as presented in Fig 3(A). Here, a circular region of microfibrosis is immediately surrounded by homogeneous tissue; 2) fibrosis with a fibrotic border where the circular region of microfibrosis is immediately surrounded by a second zone where the probability of microfibrosis decreases with the radius to zero. Outside of these two regions there is no microfibrosis, see Fig 3(C).

We run one hundred of simulations for different values of *ϕ*, see Fig 3, and calculate the probability of the different types of reentries observed in each set of simulations. We have also classified the observed reentries as *activity* or *reentry* [52], see sketches in Fig 3. The label *activity* describes the simulations that have resulted in sustained reentry but are confined to the region with microfibrosis. The label *reentry* characterizes the simulations where we observed ectopic beats leaving the microfibrosis and inducing sustained reentry all over the tissue, see a sketch in Fig 3. Simulations were monitored for 1500 *ms* and were classified as: (i) no reentry, (ii) non-sustained reentries, (iii) confined, sustained *activity* or (iv) global, sustained labeled *reentry*.

The total number of reentries in both cases are similar, compare Fig 3(B) and 3(D). However, the type of activity, confined *activity* vs. global *reentry*, varies systematically. The inclusion of a border region facilitated the transformation of confined activity to sustained, global reentry affecting also the homogeneous surrounding tissue. Therefore, we include the gradual fibrotic border in the next simulations.

### Calculation of electrocardiograms

One way to indirectly assess the degree of fibrosis in cardiac tissue is the analysis of the waveform of electrograms measured by catheters tips located near cardiac tissue during a clinical electrophysiology study. Propagation near fibrosis is associated with fractionated electrograms [31, 32]. Two-dimensional computer simulations have already given valuable insights about the relation between microfibrosis and complex fractionated electrograms [13, 15, 19].

Here, we continue these studies by also simulating electrograms (EG) measured by electrodes near the 3D tissue surface (0.1 mm from the surface). The extracellular potential is computed using the large volume conductor approximation [15]. To this end, we integrate all the currents in the tissue with respect to the position of the electrode:

$$\begin{array}{c} EG=A\int \frac{\vec{\nabla }\cdot \left(D\vec{\nabla }V\right)}{|\vec{r^{\prime }}|}d\vec{x}, \end{array}$$ (6)

where $\left|\vec{r^{\prime }}\right|$ is the distance from the electrode to the particular point of the tissue and *A* is a constant including the ratio of extracellular and the intracellular resistivities. For a list of different methods of calculations of EG see [53].

## Results

The interaction of a wave with the fibrotic region was studied in two-dimensional models [23]. A remaining activity was observed inside the fibrotic area. Under certain conditions, this activity confined to the microfibrosis region may leave and re-excite the rest of the tissue, giving rise to the generation of ectopic beats. Here, we extend those results to 3D.

### Reentry in a three-dimensional slab of tissue

**Fig 4:**
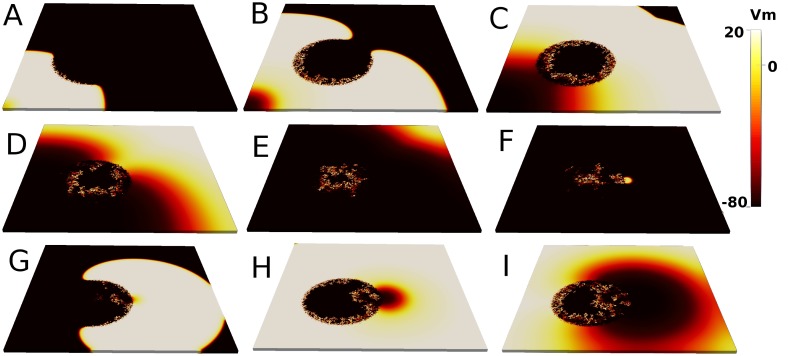
Reentry in a 3D slab of cardiac tissue. Numerical simulation showing the reentry from the fibrotic region into the whole slab of tissue. Nine snapshots of the tissue slab every *t* = 66 *ms*, therefore for times *t* = 66 *ms*, *t* = 132 *ms*, *t* = 198 *ms*, *t* = 264 *ms* *t* = 330 *ms*, *t* = 396 *ms*, *t* = 462 *ms*, *t* = 528 *ms*, and *t* = 594 *ms*. Parameter values: *ϕ* = 0.72, $\tau_{d}^{o}=0.30$, system size is 7 × 7 × 0.1 *cm*^3^ (*N* = 10 layers), radius of the fibrotic region *R*_*o*_ = 1.4 *cm*, and total time of simulation *t* = 1500 *ms*.

In Fig 4 we show a typical example of a simulation leading to reentry due to localized fibrosis in 3D. The corresponding movie is provided as supplementary material (S1 Movie). First, the wave of action potential propagates around the circular region, see Fig 4(A) and 4(B). However, the action potential slowly enters into the fibrotic region, where it breaks down into pieces, see Fig 4(C)–4(E). One of these pieces arrives to the border where it induces the formation of a new reentrant wave, see Fig 4(F)–4(G). The action potential inside the fibrotic region induces new waves into the homogeneous tissue. Therefore, via the mechanism of microreentry inside a region with microfibrosis a localized ectopic pacemaker is generated.

**Fig 5:**
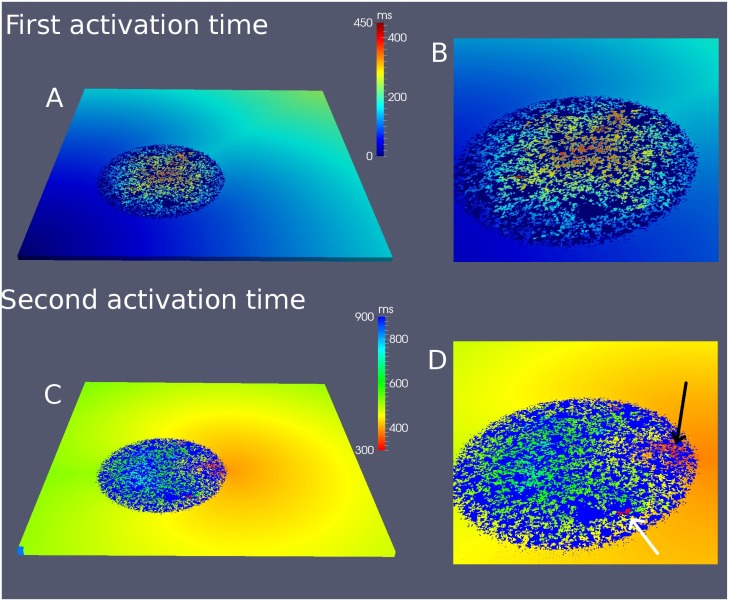
First and second activation time maps in a 3D slab of cardiac tissue. Map of the first activation of the cells corresponding to simulation shown in Fig 4 (A) and the enlargement of the map in the fibrotic area (B). Map of the second activation of the cells corresponding to simulation shown in Fig 4 (C) and the enlargement of the map in the fibrotic area (D). Black arrow shows the origin of the second activation times which produces the first reentry, and white arrow shows premature activation which dies without producing reentry.

In order to understand the process of reentry in more detail we plot in Fig 5 the maps of activation times. The time of the first activation of each cell is plotted in Fig 5(A) and 5(B). While the wave takes around 200 *ms* to arrive to the opposite corner of the system, it takes more than 400 *ms* to reach the center of the fibrotic area. Even more interesting is the time of the second activation of each cell, which only occurs in the case of a reentry. We observe in Fig 5(C) and 5(D) that the second activation occurs first near the border of the fibrotic region at 300 *ms* and it produces a propagating wave even before the center of the fibrotic region is excited for the first time at 450 *ms* (Fig 5(A) and 5(B)).

### The probability of reentry depends on the thickness

**Fig 6:**
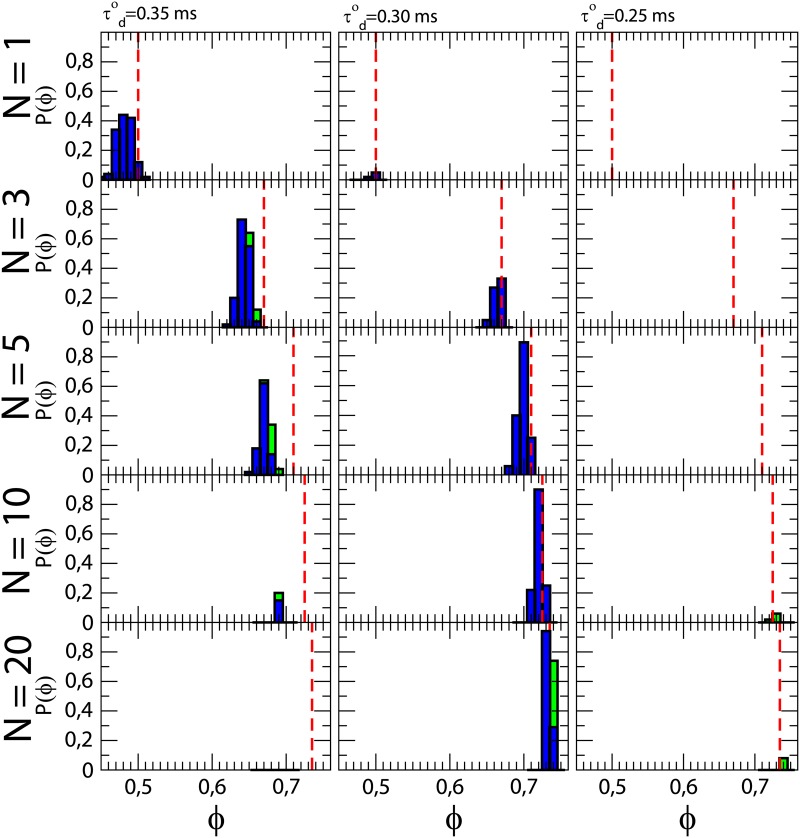
Dependence of the reentry probability on thickness and excitability. Columns correspond to three different values of the parameter $\tau_{d}^{o}$ inside the fibrotic region corresponding to three different excitabilities. Outside this region *τ*_*d*_ = 0.25*ms*. Rows correspond to five different thickness of the three-dimensional slab of tissue (*N*). For each value of $\tau_{d}^{o}$ and *N* the dependence of the probability of reentry as function of *ϕ* is plotted. For each value of *ϕ* two types of reentries are plotted: full *Reentry* (blue) and confined *Activity* (green). Dashed lines correspond to the percolation threshold value. Parameter values: system size varies from 4 × 4 × 0.01 *cm*^3^ (*N* = 1 layer) to 4 × 4 × 0.2 *cm*^3^ (*N* = 20 layers), radius of the fibrotic region *R*_*o*_ = 1.4 *cm*, and total time of simulation *t* = 1500 *ms*.

Two-dimensional studies with the same model [23] predicted the appearance of reentry for a intermediate value of *τ*_*d*_ inside the fibrotic region ($\tau_{d}^{o}$). They are summarized in the first row in Fig 6. For larger values of $\tau_{d}^{o}$ waves propagate too slow to enter into the fibrotic region (results not shown). While reentry probability arrives to 40% in two-dimensional systems with strong remodeling, only very small probability is found for $\tau_{d}^{o}=0.30$ and no reentry is found for $\tau_{d}^{o}=0.25$.

If we add two layers to the single layer employed for the two-dimensional simulations (N = 3), two important changes are observed, see second row in Fig 6. First, for strong remodeling, the whole distribution of reentries shifts, to higher values of *ϕ*, in agreement with the dependence of the percolation threshold on the thickness, see Fig 2. Second, we observe an increase of the reentry probability for intermediate and large values of $\tau_{d}^{o}$.

Next, we consider a three-dimensional slab with five layers (N = 5), see third row in Fig 6. The results for five layers confirms the shift to larger values of *ϕ* due to the change of the percolation threshold, and the further increase of the probability of reentry for intermediate values of $\tau_{d}^{o}$. In particular, for intermediate remodeling and *ϕ* = 0.7 the probability of reentry is as high as 90%. We highlight also that 5 layers correspond to 0.5 mm of thickness and that thickness is already observed in some regions of the normal atria.

The probability of reentry for $\tau_{d}^{o}=0.35ms$, is much smaller when 10 layers are employed (N = 10). On the other hand, a sizable non-zero probability for reentry for $\tau_{d}^{o}=0.30ms$, exists only in a narrow window of fractions of *ϕ*. And, more important, activity is observed already under normal remodeling with 6% of probability around the percolation threshold value. Finally, for thick slabs of tissue (N = 20), no reentry is found for strong remodeling in comparison with the high probability in two dimensional system. For intermediate remodeling the results are similar to those obtained with half of the layers. For normal remodeling there is a 8% of probability of activity again around the percolation threshold value. The increase of the thickness therefore shifts the optimal value of $\tau_{d}^{o}$ for reentry to occur to values that corresponds to intermediate remodeling, whereas in the pure 2D case, the probability was largest for strong remodeling ($\tau_{d}^{o}=0.35ms$). Altogether, the strongest occurrence of reentry does not occur for normal or strong but for intermediate remodeling. This is presumably because for normal conditions the excitation waves are more robust against perturbation by the heterogeneous distribution of available conducting links. In parallel, the tendency for rotor formation weakens as remodeling gets more pronounced.

### The probability of reentry depends on the size of the fibrotic region

**Fig 7:**
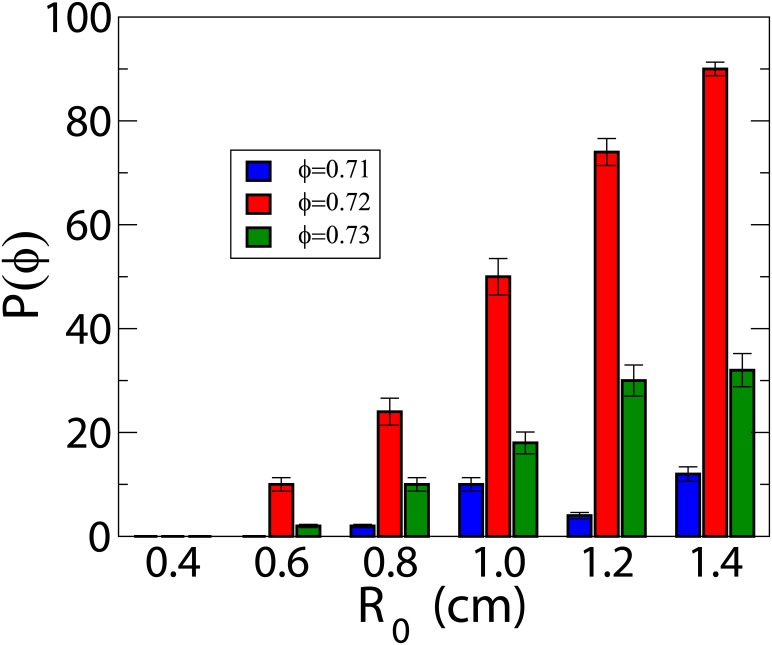
Dependence of the reentry probability on the size of the fibrotic region. For weak excitability ($\tau_{d}^{o}=0.30ms$) and thickness *N* = 10 layers we change the radius of the fibrotic region. For the radius here considered we observe reentry only for *ϕ* = 0.71, 0.72 and 0.73. The probabilities for each value of *ϕ* are plotted as function of the radius *R*_0_. Parameter values:system size is 4 × 4 × 0.1 *cm*^3^ (*N* = 10 layers), and total time of simulation *t* = 1500 *ms*.

To study the influence of the size of the heterogeneous region, we consider a set of parameter values with high probability of reentries: $\tau_{d}^{o}=0.30$ *ms* and *N* = 10, see Fig 6. In Fig 7 we show the results of these simulations, where we change the radius of the fibrotic region from 0.4 *cm* to 1.4 *cm*. The higher the size of the region is, the more likely the reentries are.

With these results we can also estimate the minimum size for a fibrotic region in a 1 *mm* thick three-dimensional system to generate reentries: this is around 1 *cm* of diameter, which is in the same order than the fibrotic spatial patterns derived from patients [54]. This value is smaller than for a pure two-dimensional system where it is around 1.5 *cm* of diameter [52].

### Simulation of fractionated electrograms in 3D

**Fig 8:**
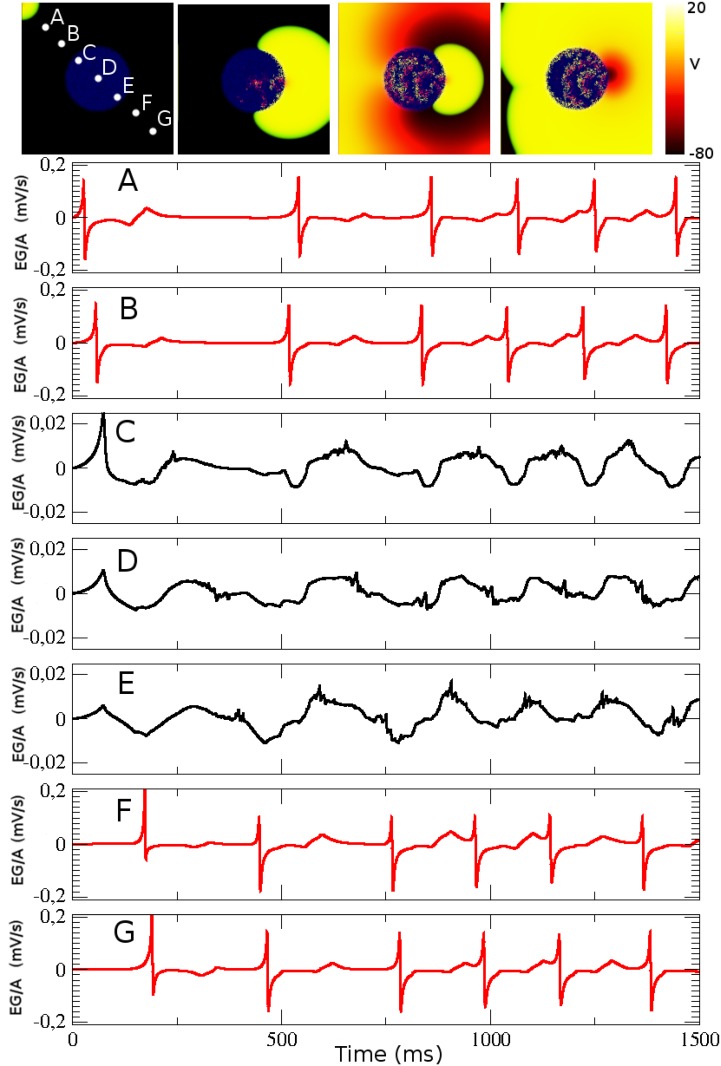
Electrograms at different locations in the tissue during reentry. The signals of different electrodes are plotted in arbitrary units for a numerical simulation showing reentry. Four snapshot of the top view of the tissue slab for times *t* = 16.5 *ms*, *t* = 500 *ms*, *t* = 1000 *ms*, and *t* = 1500 *ms*. The location of the electrodes is shown in the first snapshot by white circles. The electrodes are located at 0.01 cm from the slab of tissue. Signals calculated in each electrode, following Eq (6), are plotted. Black and red curves correspond to electrodes inside and outside of the fibrotic region, respectively. Note that vertical axis scale is modified for panels C-E due to the weak signal inside the fibrotic area. Parameter values: *ϕ* = 0.72, $\tau_{d}^{o}=0.30ms$, system size is 7 × 7 × 0.01 *cm*^3^ (*N* = 10 layers), *R*_*o*_ = 1.4 *cm*, and total time of simulation *t* = 1500 *ms*.

We consider again the 3D numerical simulation shown in Fig 4, where we choose 7 observation sites (*electrodes*), see Fig 8. The first panels of this figure correspond to four snapshots of the action potential propagation (view from the top of the surface of the 3D simulation), where the location of the electrodes are also shown.

During the simulation the EG measured by the different electrodes outside the fibrotic area are basically the same, see Fig 8(a), 8(b), 8(f) and 8(g), and equivalent to a EG measured in an homogeneous system. However, the EG measured by the three electrodes above the fibrotic region are different, see Fig 8. Inside this region the wave propagates slowly and it arrives to the middle with a large delay and pieces of waves arrive simultaneously from the border. The signals of the electrodes inside the fibrotic area are weaker, and look irregular and fractionated, see Fig 8(c)–8(e). Note that the amplitude of the EG inside the fibrotic region is weaker and the scale is one order of magnitude smaller. Actually the amplitude of the EG decreases linearly with the fraction *ϕ* due to the systematic loss of active tissue for the integral defined in Eq (6).

**Fig 9:**
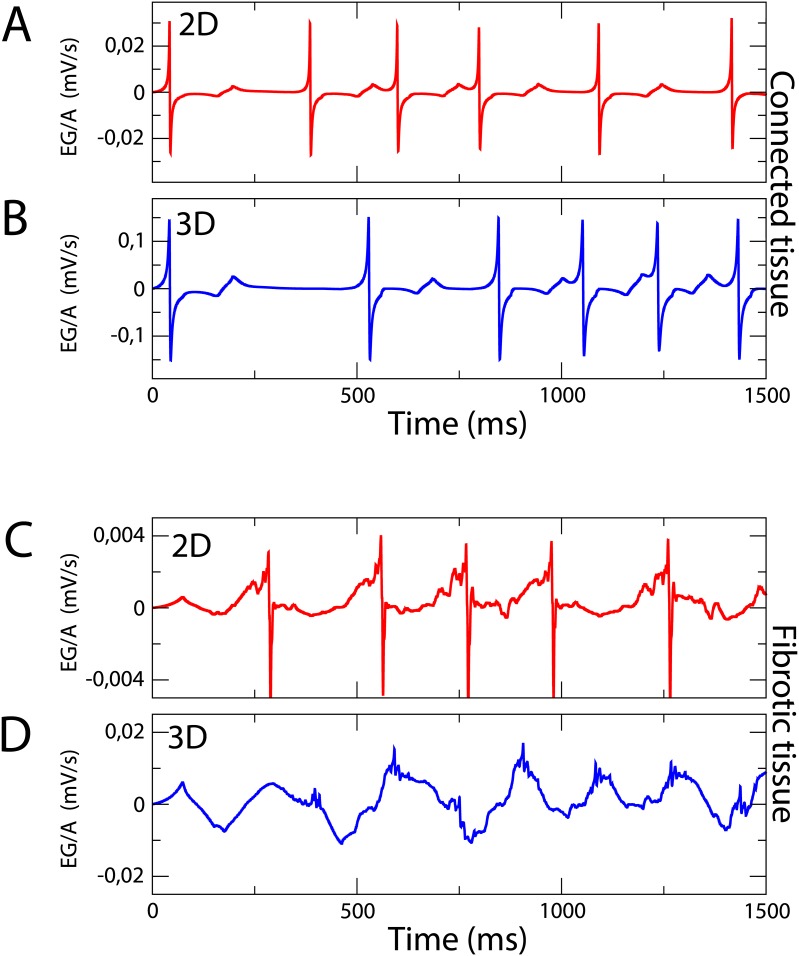
Comparison of Electrograms in 2D and 3D. Electrograms calculated on two points corresponding to locations marked with (A) and (D) in Fig 8. Two-dimensional electrograms calculated under strong remodeling conditions outside (A) and inside (C) the fibrotic area (*ϕ* = 0.48) in comparison with three-dimensional electrograms calculated under intermediate remodeling conditions outside (B) and inside (D) the fibrotic area (*ϕ* = 0.72).

Two numerically calculated EG in 2D and 3D are compared in Fig 9. They correspond to conditions where microfibrosis induces reentries. The EG measured in the homogeneous part of the tissue is basically the same in 3D and in 2D, apart from an increase in the amplitude of the EG due to the larger number of cells in 3D, see Fig 9(A) and 9(B). However the EG in the fibrotic region looks different, see Fig 9(C) and 9(D). In the 2D case the EG is already fractionated but the waveform still resembles somewhat that one of the homogeneous EG. The 3D EG measured inside the microfibrosis is much more random and very different from the homogeneous EG, probably due to an averaging effect of uncorrelated signals from different layers of the simulation. This result shows that 3D effects on the measured EGs are not negligible and should be taken into account in future studies when comparing EG in more detailed and realistic physiological models with experimentally measured CFAEs.

## Discussion

### Percolation threshold and occurrence of microreentry in microfibrosis

Our previous simulations in 2D discrete and isotropic models of cardiac tissue with simple lattice topologies (square, hexagonal) exhibited microreentry inside a fibrotic region [23]. Equivalent results were obtained with two-dimensional models with sub-cellular resolution that took into account the anisotropy of the tissue and the gap junction distribution together with a much more complete physiological description [24]. In both studies, the probability of microreentry depended on the fraction of fibrosis, *ϕ*, and it was shown to be most prevalent just above the percolation threshold *ϕ*_*c*_ where conduction is lost. Since the percolation threshold depends only on the lattice topology, details of the electrophysiological should not matter much for the identification of the *ϕ*-values for which reentry is most likely.

In this paper, 3D simulations of homogeneous tissues with an embedded region of microfibrosis confirmed again that the probability to generate reentry from a microfibrotic region is higher just above the percolation threshold for conducting links in the discrete network. Reentry is likely to appear for percentages of non-conducting links between 50% and 70%. Note that myocyte connectivity decreases to a value of 65% in high-resolution 3D reconstruction of an infarct border zone [21]. We show a straightforward relation between the size of the fibrotic region and the probability of reentry: reentries are more likely to occur inside larger-sizes of fibrotic regions. The same relation was observed before in 2D simulations [23]. One possible explanation for this observation is that the increase of the size of the microfibrosis region leads to an increase of the length of a possible reentry circuit that is formed via a zig-zag propagation in the maze. Moreover, the 3D results reported here show that there are non-trivial and non-intuitive relations between the probability of reentries, the excitability of the tissue (APD and CV) and the thickness of the tissue.

The probability of reentry depends on the remodeling of the tissue and we observe a non-monotonic behavior: probability of reentry first increases when we move from parameter values near propagation boundary (strong remodeling) to weakly excitable tissues (intermediate remodeling); then, the probability of reentry decreases when we further move from weakly excitable to normal remodeling. Non-trivial relations are also observed varying the tissue thickness. For strong remodeling, probability of reentry first increases as we increase the thickness of the tissue from a monolayer to 0.3 *mm*, but decreases as we further enlarge the thickness from 0.5 *mm* to 2 *mm*.

Source-sink mismatches produces unidirectional propagation block [55] and have an important role in the generation of the zigzag reentry [18]. The probability of source-sink mismatch increases with size and thickness. Therefore, by further increasing the thickness of the system, the source-sink mismatches are too strong and instead of generating unidirectional blocks, generate full propagation block. The fine balance between having enough driving force for wave propagation, *I*_*Na*_, and enough source-sink mismatches to generate reentry zigzag circuits explains the trends observed in the results presented in Fig 6. A similar balance or tradeoff between tissue structure and *I*_*Na*_ amplitude was also used earlier to explain slow conduction velocities observed in experiments [56–58].

### The role of fibrosis: triggering vs. perpetuating arrhythmic activity

There are several attempts to model the interaction of waves within regions of heterogeneous cardiac tissue, where cells are partially disconnected [59–61]. Their main results are a decrease of the macroscopic conduction velocity [51, 62] and a decrease of excitability which may preclude spiral breakup [26]. Related results are the observations that propagating waves perform a zigzag motion associated with the presence of non-excitable tissue [63], scars [64], propagation inside vein tissue [20] or microfibrosis [13].

However, it is worth noting that the above mentioned studies consider the role of fibrosis in the maintenance or perpetuation of arrhythmia, e. g. atrial fibrillation. To achieve this goal, the arrhythmia is always induced by premature stimulation (S1–S2 protocol) or fast pacing in the simulations. In this work, our focus is on the role of fibrosis as a trigger for arrhythmia. Our simulations present how a region with microfibrosis can sustain microreentry and become an ectopic pacemaker. For instance, Fig 8 shows that after a single stimulus the 3D tissue is rhythmically paced by the region of microfibrosis, with a cycle length around 200 *ms*. Future work may compare the outcome of different simulation protocols for a given heterogeneous setup that models regions of fibrosis inside a healthy tissue. We expect that reentry occurs earlier in the S1–S2 and fast pacing protocols than in our single-stimulus approach. Nevertheless, the parameter regions where reentry is found to be most likely to occur in our study here should also be the ones where propagation of multiple excitation waves is most vulnerable to the emergence of reentries. This is the first 3D simulations that support the hypothesis that ectopic pacemakers can be explained by the mechanism of microreentry inside a region of fibrosis. Recently, the role of fibrosis and other tissue heterogeneities in the generation of ectopic foci was also studied using different mechanisms of arrhythmia, such as trigger activity [65] and abnormal automaticity [66].

We systematically change the thickness of our 3D model. Fig 6 shows that the results are qualitatively comparable when using the monolayer or a 0.3 *mm* thick tissue. However, both the qualitative and quantitative results obtained with 0.5 *mm* to 2 *mm*-thick tissues are substantially different from the results obtained with a monolayer model indicating a crossover from genuine 2D to 3D behavior. This finding is in accordance with recent experimental and simulation-based evidence of the impact of endo-epicardial electrical dissociation during atria fibrillation [67]. During atrial fibrillation, the electrical activity of endocadial and epicardial surfaces is found to be progressively uncoupled, most likely due to interstitial fibrosis. In addition, the uncoupling is correlated with increasing stability and complexity of the atrial fibrillation substrate. Therefore, one may conclude that atrial fibrillation progressively becomes a three-dimensional process.

### Fractionated and asymmetric electrograms

Under normal conditions, surface EG show first a positive deflection, when the AP wave is arriving, followed by a negative deflection, indicating the wave is moving away. The amplitudes of these reflections are similar or symmetric. Such typical waveforms are observed, for instance, in our results presented in Fig 8. However, previous computational studies have already characterized how changes in electrogram waveform are associated with complex and fractionated propagation in the presence of fibrosis, arrhythmia, and complex 3D geometry and anisotropy [11, 13, 15, 18]. For instance, in Fig 8(f) and 8(g), the waveforms are asymmetric, with a positive deflection bigger than the following negative one. This indicates the collision of the two propagating waves, as described before in [68]. The two waves have to propagate around the fibrotic region before they collide. A negative monophasic electrogram, as in the electrode E of Fig 8(e), is usually associated with an AP focus [11, 18], since this would reflect that most of the waves propagate away for the electrode position.

Fractionated electrograms are carefully studied and related to fractionated propagation in microfibrosis in [13, 15, 18]. In our simulations we observe fractionated and complex electrograms near the fibrotic region, as presented by the waveforms in Fig 8(c)–8(e).

### Limitations, future works and conclusions

The ionic model used in the numerical simulations is simple and rather qualitative. In order to have a direct control of the parameters related with ion channels and their possible pathological or pharmacokinetic modifications, more elaborate and detailed ionic models should be employed in future studies. However previous results in two dimensions with the same model [23] were later found to be very much in line with the outcome of studies with a more realistic and physiologically detailed model [24]. Therefore we are confident that the three-dimensional results presented here should server as a good starting point and provide guidance for studies in the near future involving more realistic models incorporating detailed electrophysiology and anatomy as well as experimentally obtained characterization of fibrosis.

The geometry employed here, a three-dimensional thin slab of tissue, is relatively simple and we have already ongoing works that consider anisotropy and more accurate geometric descriptions. The number of neighboring cells in the cubic lattice employed here is six while a more realistic organization of the cells is probably closer to a three-dimensional close-packed hexagonal organization in which each cell has twelve nearest neighbors. Since the percolation threshold in such a lattice is substantially larger than the one in cubic lattice, reentries will also occur at a much larger value of *ϕ* in the hexagonal lattice than in the cubic lattice.

The development of more elaborate descriptions of heterogeneous cardiac tissue including specific information of this type is an actual research direction of several groups with a lot of implications in cardiac modeling for the correct description of fibrotic regions in cardiac tissue. For example, reactive interstitial fibrosis has been modeled by introducing non-conductive materials that disconnect a fraction *ϕ* of neighboring myocytes, however, fibroblast are also frequently present in such regions which can be electrically coupled to myocytes and affect their properties [43, 69–72].

In summary, we find that a confined region with microfibrosis may generate microreentries. Such active regions may act as ectopic pacemakers for the surrounding tissue, exhibit the electrogram signature of CFAEs and are intimately linked to the fraction of fibrosis, *ϕ*, and topology of connections between myocytes. The essential parameter characterizing the topology of the lattice of discrete nodes in the simulation (or the network of cells in experiments) is the associated percolation threshold *ϕ*_*c*_. The phenomena reported here: microreentry inside fibrotic regions, macroreentry and ectopic pacemakers triggered from fibrotic areas and the associated complex fraction electrograms all occur if the fraction of broken links representing the degree of fibrosis is such that the network of the remaining conducting links is just above the percolation threshold.

## Supporting Information

S1 Movie Reentry in a 3D slab of cardiac tissue. Numerical simulation showing the reentry from the fibrotic region into the whole slab of tissue.
(AVI)
